# Non-Obscene Socially Inappropriate Behavior in Patients with Gilles de la Tourette Syndrome

**DOI:** 10.3390/jcm13195926

**Published:** 2024-10-04

**Authors:** Mateusz Grycz, Piotr Janik

**Affiliations:** Department of Neurology, Medical University of Warsaw, 02-091 Warsaw, Poland; mateusz.grycz@uckwum.pl

**Keywords:** non-obscene socially inappropriate behavior, Tourette syndrome, coprophenomena, attention deficit hyperactivity disorder, oppositional defiant disorder, autism spectrum disorder, anxiety disorders

## Abstract

**Background/Objectives**: Behavioral disturbances are a common phenomenon associated with Gilles de la Tourette syndrome (GTS), which can manifest as non-obscene socially inappropriate behaviors (NOSIBs). The classification of NOSIB has not yet been clearly established. The objective of this study was to determine the frequency, age of onset, and clinical correlation of NOSIB with tic severity and the prevalence of comorbid psychiatric disorders in individuals with GTS. **Methods**: A total of 365 participants (272 male, 74.5%) with GTS were included in the study. Of these, 278 (76.2%) were children and adolescents. The mean age of the participants at evaluation was 14.4 ± 9.8 years, with a range of 4 to 64 years. The clinical data of NOSIB were collected during a routine, ambulatory examination using half-structured questionnaires developed by the authors. **Results**: NOSIB was observed in 86 patients with GTS, representing a prevalence of 23.6%. NOSIB commenced at a mean age of 6.6 ± 4.1 years (range 2–19). The mean age at onset of NOSIB was 1.4 ± 3.7 years after the onset of tics, with 18 cases (26.1%) preceding tics and 13 cases (18.8%) starting at the same age as tics. The results of the multivariate analysis confirmed the associations between NOSIB and YGTSS (*p* = 0.02) and coprophenomena (*p* < 0.01), as well as ADHD (*p* < 0.01), ODD (*p* = 0.01), ASD (*p* < 0.01), and anxiety disorders (*p* = 0.02). **Conclusions**: NOSIB is an early symptom of GTS that typically manifests in childhood and occurs in approximately a quarter of patients. Tic severity and the presence of psychiatric comorbidities, which indicate a more severe disease course, may serve as risk factors for NOSIB.

## 1. Introduction

Gilles de la Tourette syndrome (GTS) is a complex neuropsychiatric condition that is characterized by the presence of motor and vocal tics [1]. As defined in the *Diagnostic and Statistical Manual of Mental Disorders, Fifth Edition* (DSM-5), tics are characterized as sudden, rapid, recurrent, non-rhythmic movements or vocalizations that exhibit waxing and waning frequency, intensity, number, complexity, and type [2]. The diagnostic criteria necessitate the presence of a minimum of two motor and one vocal tic at any point during the course of the illness for a period exceeding one year, with an onset prior to the age of 18 years [3]. The disease typically manifests during childhood, with a peak incidence at approximately five or six years of age. GTS is often accompanied by socially inappropriate behavioral issues that may be indicative of an underlying condition, such as attention deficit hyperactivity disorder (ADHD) or oppositional defiant disorder (ODD) [4]. Nevertheless, behavioral issues are not always a consequence of comorbid psychiatric disorder. Socially inappropriate behaviors may be further classified as obscene or non-obscene. Obscene behaviors are those resulting from complex tics, such as coprolalia and copropraxia (coprophenomena). Non-obscene socially inappropriate behaviors (NOSIBs), which are not a distinct disease entity, encompass actions that could be indicative of a range of psychiatric disorders. The classification of NOSIB in GTS patients has been a topic of debate. Previous studies have observed these behaviors in GTS patients [5,6], yet their categorization as a comorbid psychiatric disorder, a complex tic, or a spectrum of these disorders remains a challenge. In contrast to obscene socially inappropriate behaviors, NOSIB has not been extensively studied in GTS. Currently, there are only two studies dedicated to NOSIB in GTS [5,6].

The term “non-obscene, socially inappropriate behavior” was first introduced in the context of GTS by Kurlan et al. [5]. Non-obscene, socially inappropriate behavior (NOSIB) was defined as any action or conduct that is deemed inappropriate or unacceptable within a given social context and is challenging or impossible to control. In their study, a self-report questionnaire was administered during an outpatient visit. A subsequent study [6] employed a similar self-report methodology to ascertain whether specific characteristics predict the urge to perform socially inappropriate behaviors. The two aforementioned studies yielded disparate results with regard to the incidence of NOSIB in GTS, with a range of 22–60%. The relatively modest sample sizes (*n* < 100) have presented a significant challenge in identifying clinical correlates between NOSIB and comorbid conditions. In one study, a control group without NOSIB was not included in the analysis [5], while in the other, the non-NOSIB group was limited to only 20 patients [6]. Moreover, the assessment of all comorbid psychiatric disorders in GTS was not conducted with the objective of determining their correlation with NOSIB. While a positive correlation between obsessions and the presence of NOSIB has been identified, the rates of obsessive compulsive disorder (OCD), depression, anxiety, and ODD in NOSIB patients remain under-researched [6]. To date, studies have only examined subjects older than 14 years, despite the onset of tics occurring at a much younger age (mean 5 years) and the peak severity occurring in late childhood or early adolescence, typically between 10 and 12 years [7]. In light of these considerations, the use of self-administered questionnaires may introduce substantial recall bias. Furthermore, there is a lack of information regarding the verification of patient responses during face-to-face assessments with clinicians, which could be crucial in differentiating between mislabeled attention or conduct issues and actual NOSIB.

The relationship between NOSIB and emotional symptoms, conduct issues, hyperactivity/inattention, and risky decision-making was examined in a large group (*n* = 1280) of 14-year-old adolescents (not screened for GTS) born across the UK [8]. In that study, NOSIBs were defined as behaviors such as being rude or noisy in public or misbehaving in lessons, and were scored based on the frequency of occurrence as reported by the subjects. The findings indicated that all forms of NOSIB were associated with hyperactivity and conduct problems. However, only misbehaving in the classroom was linked to risky decision-making. As NOSIB could be identified as either conduct or attention issues using the aforementioned definition, the authors proposed that a more standardized definition of NOSIB was necessary to classify NOSIB as an independent phenomenon.

The present study employs a large sample of consecutive GTS patients to establish the incidence and clinical correlates of NOSIB in a real-life clinical setting. It is hypothesized that (1) NOSIBs manifest at an early age, as evidenced by the correlation between NOSIB and ADHD, which is more prevalent in that age group; (2) the clinical correlation between comorbid psychiatric conditions and NOSIB differs when assessed in a large sample; (3) GTS patients with autism spectrum disorder (ASD) experience NOSIB more frequently, due to their social cognitive impairment; (4) NOSIB are more common in GTS patients experiencing more severe tics and more frequent comorbid psychiatric disorders.

## 2. Materials and Methods

All patients were recruited from a single outpatient clinic led by a clinician with expertise in tic disorders (PJ). Patients were only enrolled in the study once they met the diagnostic criteria for GTS. This study was designed as a one-time registration study, with patients entered into a database on a single visit. No follow-up data were included in the analysis. The collection of clinical data from patients with GTS was approved by the Ethics Committee of the Medical University of Warsaw (KB/2/2007).

Patients were evaluated for the diagnosis of GTS in accordance with the criteria set forth in the *Diagnostic and Statistical Manual of Mental Disorders* (DSM-5). A semi-structured interview was conducted with the aid of a questionnaire based on the Tourette Syndrome International Database Consortium Data Entry Form, developed by Freeman et al. [9], which included demographic data as well as the presence of most common comorbid disorders encountered in GTS such as ADHD, ODD, CD, OCD, depression, anxiety disorders. Each patient was subjected to a meticulous examination to ascertain the presence of symptoms enumerated in the DSM-5 diagnostic criteria for the aforementioned disorders. The diagnoses of mental disorders previously established by a psychiatrist prior to the evaluation were factored into the analysis.

The Yale Global Tic Severity Scale (YGTSS) was utilized to assess the tics experienced by patients within the week preceding their evaluation. The questionnaire developed by Kurlan, which was designed to primarily characterize NOSIB [5], was not utilized in this study, as our objective was not to ascertain the characteristics or subdivisions of NOSIB. Our investigation was instead focused on the presence or absence of NOSIB in a given individual, with the objective of determining the prevalence and clinical correlates of NOSIB in GTS. To this end, we devised a brief semi-structured interview format to gather data on NOSIB, incorporating illustrative examples of vocal and physical actions (see Appendix A). Some of these behaviors were derived from the questionnaire developed by Kurlan et al. [5]. Only repetitive and unprovoked inappropriate behaviors were registered. Single episodes that would not otherwise be reported by the parents or patients themselves were not included in the analysis. Additionally, behaviors that were secondary to refusal or lack of reward (e.g., inappropriate behavior in response to a computer game ban) or better explained by a different phenomenon (e.g., breaking toys due to an anger outburst, unacceptable/inappropriate touching of others as a part of a complex motor tic or compulsion) were excluded. Ultimately, we concluded that a face-to-face interview would be more effective than a self-report questionnaire in achieving the objectives of our study. The category of dangerous behaviors, defined as potentially leading to direct physical harm (e.g., sticking one’s head outside a vehicle window, running onto a busy road, or touching hot objects), with possibility of inflicting self-harm, were not socially inappropriate with the context of NOSIB and therefore not deemed to fall within the scope of NOSIB. Similarly, behaviors that could be considered unusual but not necessarily misbehavior (e.g., asking nonsensical questions, such as “Does soup have eyes?”) were excluded. This differentiation was made in order to align more closely with the criteria set out by Kurlan et al. [5] and to create a more cohesive group.

Information regarding NOSIB was gathered from children and adolescents, as well as their parents. For younger children and individuals with severe ASD, clinical data were primarily obtained from parents. Adult patients primarily self-reported their symptoms.

In cases where NOSIB was present, a more detailed examination was conducted to ascertain its characteristics, including the age of onset, the presence of the behavior at the time of evaluation, and consequences of the misbehavior. Additionally, the patients were queried as to whether they deliberately avoided situations that could potentially precipitate NOSIB or feared that the symptoms might recur. We also inquired as to whether they perceived the symptoms as a disease entity or as a manifestation of their own personality (egodystonic vs. egosyntonic feature). Finally, patients were asked whether performing the NOSIB action brought them a sense of relief (see Appendix A).

The cohort of patients assessed for the presence of NOSIB included 365 consecutive patients seen at the clinic between 2017 and 2024. The group was predominantly male (272, 75%). The age at the time of assessment ranged from 4 to 63 years (mean 14.4, SD 9.8). A total of 87 adults (23.8%, range 18–63, mean 28.8, SD 10.2) and 278 children and adolescents (76.2%, range 4–17, mean 9.9, SD 3.0) were evaluated. The mean age at onset of GTS was 5.8 years (range 2–15, SD 2.3) and the mean duration of the disease was 6.9 years (range 1–43, SD 6.8).

Statistical analyses were performed using RStudio 2023.12.1+402 "Ocean Storm" Release for Windows (© 2009–2023 Posit Software, PBC). Collected quantitative data were presented as the arithmetic mean and standard deviation, discrete numerical variables were shown as medians and quartiles (Q1, Q3), and categorical variables as absolute numbers with frequencies expressed as percentages. Comparisons between two independent groups were made using one-way analysis of variance without replication or the Mann–Whitney U test. This particular test was selected due to its non-parametric nature, in contrast to the *t*-test, which assumes a normal distribution of the underlying data. However, this assumption may not always be valid. Qualitative data were compared using the chi-squared test of independence. Variables that were statistically significant in the univariate analysis were entered into the logistic regression model to determine predictors of NOSIB in GTS patients. The results of the analyses were considered significant if a two-tailed test yielded *p* < 0.05. Gender and age were included as control variables in the multivariate regression model.

## 3. Results

### 3.1. NOSIB Prevalence

NOSIB was identified in the course of the study as having occurred during the lifetime of 86 (23.6%) of the participants, who were aged between 4 and 40 years at the time of evaluation. The NOSIB group was predominantly male (*n* = 69, 80.2%), with a male-to-female ratio of approximately 4:1 (Table 1). NOSIB manifested at some point in 19 of 87 adults (21.8%) and 67 of 278 children and adolescents (24.1%).

### 3.2. NOSIB Duration

The duration of GTS was comparable between the NOSIB(+) and NOSIB(−) groups. Of the 82 patients, 68 (82.9%) were still experiencing NOSIB at the time of the evaluation, while the remaining patients only reported a history of NOSIB in the past, which means that NOSIB resolved in 14 patients (17.1%) at the time of examination. Of these, five were adults, four were adolescents, and five were children.

### 3.3. NOSIB Onset

Information on the age of onset of NOSIB was available for 70 patients, with a mean of 6.6 years (SD 4.1, range 2–19 years) and a median of 5 years. The onset occurred most commonly in early childhood, with 58 cases (82.9%) starting at or below the age of 10 years. The duration of NOSIB from onset to evaluation ranged from 0 to 21 years, with a mean of 4.1 years (SD 4.4). The group with unknown NOSIB onset was older on average and comprised 12 adults and 4 patients under the age of 18. Information on the age of NOSIB onset, together with the onset of tics in general, was available for 69 patients. Within this group, NOSIB commenced, on average, 1.4 years (SD 3.7, range: −7–12 years) after tic onset. Of the cases, 18 (26.1%) commenced before tics, while 13 (18.8%) started at the same age as tics.

### 3.4. NOSIB Features

Information regarding the sense of relief following NOSIB and NOSIB intensity was available for 82 subjects. Two patients (2.4%) exhibited an increase in the sense of tension, while 10 patients (12.2%) demonstrated a decrease. In 31 patients (37.8%), NOSIB had no effect on the sense of tension. Around half of the patients (39, 47.6%), mainly young children (mean age 10.2 years, SD 6.7) who were not aware of their misbehavior, were unable to discern whether NOSIB brought any relief.

### 3.5. NOSIB Consequences, Suppressibility and Awareness

In approximately 50% of cases (41 out of 82 patients), NOSIB had no discernible consequences. The data indicated that minor and major consequences occurred in 33 (40.2%) and 3 (3.7%) cases, respectively. Only five patients (6.1%) were able to suppress NOSIB action once the urge occurred. In total, 6 out of 82 patients (7.3%) were actively avoiding situations that provoke NOSIB. In addition, 9 out of 82 patients (11%) in the NOSIB group believed that the inappropriate behaviors were a symptom of a disease, and 11 out of 82 patients (13.4%) were afraid that they would recur.

### 3.6. NOSIB Correlation with Psychiatric Disorders

The univariate analysis revealed a significant correlation between NOSIB and YGTSS, the presence of coprophenomena, ADHD, OCD, anxiety disorders, ODD, and ASD (Table 1). The multivariate logistic regression analysis confirmed a significant association between NOSIB and YGTSS, the presence of coprophenomena, ADHD, anxiety disorders, ODD, and ASD (Table 2).

## 4. Discussion

Our findings revealed that one in five GTS patients exhibited behaviors that could not be clearly attributed to any known psychiatric disorder. It remains unclear whether NOSIB should be considered a distinct entity. However, this disturbance may contribute to the behavioral issues commonly observed in GTS patients [4]. The onset of NOSIB occurs at an early age and is closely correlated with the onset of tics, with approximately 45% of patients experiencing NOSIB at or even before the onset of tics. It may be hypothesized that NOSIBs are under recognized as the initial symptoms of GTS, as they are often misattributed to behavioral issues or disregarded entirely. It is noteworthy that the onset of NOSIB was unknown for 16 patients. The lack of information on age of NOSIB onset could be attributed to recall bias, given that the majority of this group were adults and that NOSIB typically manifests at an early age. The duration of the disease was not a statistically significant factor in the development of NOSIB, which is consistent with the early onset of this condition.

The majority of cases included in our sample exhibited the presence of NOSIB at the time of evaluation, which suggests that NOSIB may be a contributing factor in the decision to seek medical assistance. However, it is possible that NOSIB may have a fluctuating course in at least some patients, given that in one-fifth of patients, NOSIB occurred only in the past, similar to tics and behavioral problems related to ODD and ADHD. As this was a one-time registration study, it was not possible to monitor the resolution nor the appearance of symptoms in NOSIB-diagnosed or NOSIB-free subjects, respectively. It is therefore recommended that a longitudinal study be conducted to answer the question of the age at which NOSIB symptoms may subside, if at all, and in how many patients the condition will last into adulthood.

Our findings revealed that patients with NOSIB exhibited more severe tics in general and a higher prevalence of comorbid psychiatric disorders (see Table 1 and Table 2). More intense tics are often associated with a higher prevalence of psychopathology and social disinhibition, which may in turn increase the risk of misbehavior resulting from NOSIB [4]. This suggests that the presence of NOSIB is indicative of a more severe course of GTS, which may in turn affect the severity of tics and comorbid conditions. The feeling of relief that follows the execution of a tic and the ability to suppress it are hallmarks of tics. Our findings of lack of relief of tension after NOSIB and inability to suppress NOSIB in the majority of cases suggest that NOSIB may not belong to the spectrum of tics. On the other hand, similarly to tics, approximately 90% of patients did not perceive NOSIB as a disease, considering those symptoms to be egosyntonic, despite the fact that they are exaggerated and deemed abnormal. Additionally, NOSIB occurred four times more frequently in males compared to females, which is the same gender proportion as for tics in GTS.

As demonstrated in Table 1 and Table 2, the presence of coprophenomena was found to be significantly associated with NOSIB. Our findings provide further evidence to support the previously established correlation between NOSIB and urges to use obscene language [10]. Given the discrepancy in the onset of coprophenomena and NOSIB, with the former rarely manifesting in the first year of the disease and occurring later in life in general (10 vs. 7 years, respectively), it can be postulated that NOSIB may be a risk factor for coprophenomena, rather than the other way around. Furthermore, coprophenomena were previously found to be associated with the presence of behavioral symptoms, which could have actually been a manifestation of NOSIB [11]. It is well established that coprophenomena significantly impact the quality of life of GTS patients, including family life and school/work activities [12,13]. When associated with concurrent NOSIB, coprophenomena could further negatively influence patients’ daily living and contribute to the social stigmatization and withdrawal.

Our study was the first to demonstrate a correlation between ODD and NOSIB. This would be consistent with the previously identified correlation between CD and NOSIB [5,6,8]. Although CD and ODD are not synonymous, they are conceptualized as falling within the same psychopathological spectrum of disruptive behavior disorders [14]. The greater prevalence of ODD in this group may be attributed to the tendency of ODD patients to exhibit greater levels of uncooperativeness, defiance, and hostility towards others. Such characteristics have the potential to result in socially inappropriate behaviors. Furthermore, our study confirmed the higher prevalence of ADHD in the NOSIB group, a finding that aligns with previous research [5,6]. Given the association between ADHD and disruptive behaviors, the relationship of ADHD with NOSIB appears to be a logical conclusion. Furthermore, the onset of ADHD symptoms typically precedes the emergence of tics, a phenomenon that has been observed in our study as well with regard to NOSIB. This suggests a potential association between NOSIB and ADHD. As with ADHD and other disruptive behaviors, NOSIB could be defined as an enduring pattern of inappropriate behavior. When the results from all three papers on NOSIB in GTS are considered collectively, it can be concluded that NOSIB is strongly related to disruptive behavior disorders.

Those with autism spectrum disorder (ASD) demonstrate persistent deficits in social communication and social interaction across multiple domains. This suggests that they are unable to accurately discern the social context of their actions. NOSIB may also result from difficulties in adjusting behavior to suit various social contexts. This is substantiated by the statistically significant correlation between ASD and NOSIB, as evidenced by our multivariate analysis (see Table 2). Additional support for this association can be found in a study on functional MRI during theory of mind tasks of GTS patients, which demonstrated that the presence of multiple forms of NOSIB correlated with activity within the left temporoparietal junction [15]. This region has been previously shown to play a crucial role in reasoning about others’ beliefs or inferring their intentions or desires, a skill that is often impaired in ASD patients [16].

We hypothesize that the correlation between NOSIB and anxiety disorders stems from the fact that they are a component of a collective psychopathology leading to a greater prevalence of comorbid psychiatric conditions, and therefore a more severe GTS course. However, caution is advised in such interpretations, given the correlation of NOSIB and OCD, which had been previously classified as part of anxiety disorders, as observed in the two previous studies [5,6] as well as partially in ours, albeit only in univariate analysis. This argument remains an open question.

The hypothesis that clinical correlations between NOSIB and psychiatric conditions differ when assessed on a larger sample was only partially confirmed. Our findings align with previous studies [5,6] in substantiating the existence of an association between NOSIB and hyperactivity/attention problems, obsessions, coprophenomena and conduct issues. Furthermore, our results extend this association to include ODD and anxiety disorders.

The findings of our study indicated that approximately 90% of patients did not express fear or had no opinion toward the recurrence of symptoms and did not actively avoid situations that could potentially elicit NOSIB. This is surprising given the association between NOSIB and anxiety disorders as described above. It seems that inappropriate behaviors are not linked to phobic anxiety disorders as the avoidance of phobic situations is not a defining feature of NOSIB. One potential explanation for the lack of fear of NOSIB is the absence of significant consequences associated with those behaviors, as seen in our findings. An alternative hypothesis is that NOSIB patients tend to perceive these behaviors as part of their personality, despite the fact that they are exaggerated and deemed abnormal by society. This deficit in social perception in our patients is supported by the observation of a greater prevalence of social disinhibition and poor impulse control in patients diagnosed with GTS [17,18].

Psychosocial interventions appear to be the primary treatment for children with NOSIB. The treatment plan should involve the patient, their family, the school, and the wider community, depending on whether the behavior in question occurs in a specific context or is pervasive in multiple settings. The principal objective of the intervention should be to foster awareness among children of the implications of their actions in specific social contexts, thereby facilitating the development of a more nuanced understanding of their conduct. A training programme for parents should equip them with the ability to identify problematic behaviors and apply an appropriate form of punishment or reinforcement in order to decrease their child’s unwanted behaviors and promote prosocial behaviors. The objective of functional family therapy is to identify factors within the family environment that may contribute to the emergence of socially inappropriate behaviors. In cases where NOSIB is predominantly manifested within the educational setting, it is recommended that school-based interventions be employed with the objective of enhancing academic performance and fostering positive peer relationships. This approach should be complemented by the active involvement of educators to prevent the emergence of antisocial behaviors. It is therefore recommended that psychological, educational and social interventions be included in a bespoke psychotherapy programme designed to address the specific symptoms of NOSIB. Furthermore, comorbidities such as ADHD, ODD, and anxiety should be identified and treated. A study on the impact of tic therapies and specific comorbid psychiatric conditions on NOSIB resolution would be highly informative, as it could potentially provide a targeted NOSIB strategy, similar to the use of antipsychotics and clonidine, which have been demonstrated to be effective in reducing not only tics but also behavioral disturbances. It is proposed that the recognition of NOSIB-type behaviors has the potential to inform the choice of different behavioral interventions in GTS and influence the decision to initiate pharmacological treatments for tics and co-morbid conditions.

## 5. Limitations

The present study is not without limitations. Firstly, the lack of follow-up data precludes the possibility of demonstrating NOSIB progression or resolution over time. Secondly, the current therapy of GTS was not included in the data set, thus preventing an investigation of its potential role in NOSIB severity. Lastly, the patients were referred to a neurologist and those with more problematic behavioral issues and more severe psychopathology would be sent to psychiatric clinics, which might result in a lower prevalence of NOSIB and comorbid psychiatric problems.

## 6. Conclusions

NOSIB is an early symptom of GTS that manifests in childhood and is experienced by approximately one in four patients. In some cases, NOSIB may precede the onset of tics. The correlates of NOSIB include tic severity, disruptive behavior disorders, ASD and anxiety disorders.

## Figures and Tables

**Table 1:** Univariate comparisons between NOSIB-present and NOSIB-absent groups (*n* = 365, missing data were deleted case-wise).

Variable	NOSIB Present (*n* = 86)	NOSIB Absent (*n* = 279)	*p*-Value
Gender (females), n (%)	17 (19.8)	76 (27.2)	0.266
Age at evaluation (years), mean (SD)	13.4 (8.5)	14.7 (10.1)	0.567
Disease duration (years), mean (SD)	6.9 (6.7)	6.9 (6.9)	0.789
**Coprophenomena, n (%)**	**33 (38.4)**	**33 (11.8)**	**0.001**
**YGTSS, median (Q1–Q3), min.–max.**	**47 (35–63),** **10–91**	**36 (20–52), ** **7–98**	**<0.001**
**ADHD, n (%)**	**34 (40.0)**	**27 (9.7)**	**<0.001**
**OCD, n (%)**	**22 (25.6)**	**30 (10.8)**	**0.003**
**Anxiety disorders, n (%)**	**49 (57.0)**	**109 (39.1)**	**0.024**
Depression, n (%)	15 (17.4)	32 (11.5)	0.100
**ODD, n (%)**	**26 (30.2)**	**16 (5.7)**	**<0.001**
**ASD, n (%)**	**18 (20.9)**	**7 (2.5)**	**<0.001**

NOSIB, non-obscene socially inappropriate behavior; n, number; SD, standard deviation; YGTSS, Yale Global Tic Severity Score; Q1, lower quartile; Q3, upper quartile; ADHD, attention deficit hyperactivity disorder; OCD, obsessive compulsive disorder; ODD, oppositional defiant disorder; ASD, autism spectrum disorder. Bold values are those statistically significant (*p* < 0.05).

**Table 2:** Multivariate logistic regression estimates (*n* = 365).

Variable	OR	95% CI	*p*-Value
Gender (females)	0.75	−1.06–0.44	0.448
Age at evaluation	0.98	−0.07–0.02	0.339
**YGTSS**	**1.02**	**0.003–0.04**	**0.024**
**Coprophenomena**	**3.26**	**0.48–1.89**	**0.001**
**ADHD**	**3.19**	**0.40–1.92**	**0.003**
OCD	1.66	−0.33–1.33	0.229
**Anxiety disorders**	**2.08**	**0.10–1.38**	**0.024**
**ODD**	**3.58**	**0.38–2.18**	**0.005**
**ASD**	**10.9**	**1.34–3.55**	**<0.001**

n, number; OR, odds ratio; CI, confidence interval; YGTSS, Yale Global Tic Severity Score; ADHD, attention deficit hyperactivity disorder; OCD, obsessive compulsive disorder; ODD, oppositional defiant disorder; ASD, autism spectrum disorder. Bold values are those statistically significant (*p* < 0.05).

## Data Availability

The raw data supporting the conclusions of this article will be made available by the authors on request.

## Appendix A

Non-obscene socially inappropriate behaviors (NOSIBs):

Examples of NOSIB:

- Derogatory or inappropriate remarks about people (family member, friend, stranger):
  ○ Talks to adults on a first name basis;
  ○ Makes inappropriate remarks to another person regarding their weight (e.g., “How fat you are”);
  ○ Makes inappropriate remarks about height (“You tiny guy!”);
  ○ Makes inappropriate remarks about appearance (“How ugly you are”, “You have: a big nose/crooked legs/massive breasts”) and surnames (“Hart–fart!”);
  ○ Makes inappropriate remarks about intelligence (“You idiot”) and smell (“You smell”).
- Improper actions against people (family member, friend, stranger):
  ○ Shouts or wants to shout in situations requiring silence (e.g., church, library, lesson);
  ○ Laughs or barely holds in laughter in situations requiring seriousness (funeral of a close person, prayer for the dead, accident in which a car hits a person); makes silly faces or sniggers without reason in an inadequate situation causing surprise/embarrassment of those present;
  ○ Calls an adult and schedules a meeting despite being a child;
  ○ Shouts “Fire”, “Bomb” and “I have a gun” in public places;
  ○ Turns off the TV while others are watching; throws object and/or destroys various objects;
  ○ Unable to keep their distance, e.g., leans forward almost touching another person’s face and touches other people causing their surprise/embarrassment;
  ○ School: goes under the desk in class; tries to hug/kiss the teacher;
  ○ Sexual: puts their hands in underwear, takes the underwear/trousers off in the presence of other people, undresses and shows off their genitals to others and touches breasts or other body parts of adult women/men.

Onset:

Current: Yes/No

Tension: Reduction–Increase–No impact

NOSIB intensity:

1. Urge without action;
2. Action started without completion;
3. Action completed;
4. Minor consequences of action;
5. Major consequences of action requiring medical intervention.

Do you purposely avoid situations leading to the behavior? Yes–No–Do not Know

Do you consider the behavior a sign of a disease?       Yes–No–Do not Know

Are you afraid that the behavior will occur again?      Yes–No–Do not Know
